# On the potential for crop yield increases to spare land: the role of rebound effects

**DOI:** 10.1098/rsos.260431

**Published:** 2026-10-07

**Authors:** Nelson B. Villoria

**Affiliations:** Department of Agricultural Economics, Kansas State University, Manhattan, KS, USA

**Keywords:** land sparing, rebound effects, Jevons effect, demand elasticity, agricultural productivity, total factor productivity, crop-land expansion, deforestation, international trade, technology spill overs

## Abstract

This essay discusses the economic mechanisms determining when yield growth spares land and when it drives expansion. The source of growth matters: technical change raises output with a fixed set of inputs, lowering production costs, whereas intensification requires more inputs and, unless those inputs are themselves more productive, raises costs. Because lower costs translate into lower prices, the outcome turns on how consumption responds. Where demand is inelastic, so that consumption changes less than proportionally to price, productivity gains lower farm profits and spare land, although rebound effects mean sparing is rarely complete. Where demand is elastic, gains raise profitability and cultivated area expands if land is available (backfire or Jevons effect). Farmers in agricultural frontiers supplying large domestic and international markets face elastic demand, making such regions prone to backfire. Globally, however, demand for agricultural output has been historically inelastic. This fact, combined with vigorous growth in global agricultural productivity, has resulted in net global land sparing. A slowdown in total factor productivity growth over the last decade and a half threatens these gains. Sustaining this outcome requires renewed investment in agricultural research and development, and policies that constrain frontier expansion.

## 1. Introduction

One of the most important achievements of the global food system in the recent past was increasing crop yields, so it has been possible to feed a population that more than doubled in size and became richer, while using virtually the same absolute amount of land [1–4].

Between the early 1960s and mid-2010s, global crop production tripled while the total agricultural land area (crop-land plus permanent meadows and pastures) expanded by only 11% [4]. During the same period, the area harvested for cereals grew by less than 2% [5]. Although the more recent evidence points to a moderate expansion of crop-land, around 80 million hectares (Mha), or 5% since 2001 [6], this has been more than offset by a loss of about 150 Mha of permanent meadows and pastures, so that the total agricultural land base has remained roughly stable or slightly contracted [6].

Meanwhile, population growth has continued to outpace crop-land expansion so that the global average crop-land area needed to feed, clothe and provide biofuels for a single person has decreased significantly. This continues the trend documented by Ramankutty *et al.* [7], who found a drop from 0.76 ha per capita in 1900 to 0.35 ha per capita by 1990, driven by improved agricultural productivity. This decline continued into the twenty-first century, with global crop-land per capita falling 37.5% from 0.347 ha in 1985 to 0.217 ha in 2022 [8].

These global averages, however, mask significant spatial heterogeneity in how technical change affects land use across the world. While the global agricultural land base appears stable, it is the result of countervailing regional extremes of contraction and expansion. Significant land abandonment or conversion to permanent pastures has been observed in the northern Great Plains of Canada and northern Russia, as well as in parts of China where former crop-lands are being replaced by tree plantations, orchards and urban areas [9]. By contrast, crop-land expansion continues to occur in South America—specifically across Brazil, Argentina, Paraguay, Bolivia and Uruguay—and within Sahelian and Central African nations [9].

The fact that global crop production grew on a nearly constant land base is primarily owing to higher yields. Blomqvist *et al.* [10] decomposed the sources of growth in agricultural production, showing that between 1961 and 2015 global crop production increased by 250%, and 89% of this increase came from aggregate yield growth (output per unit area per year). Moreover, increases in cropping intensity (number of crops grown in the same growing season) explain 23% of the overall increase in aggregate yield in the same period.

## 2. Yield growth, technology and productivity

The dominance of yield growth points to the central role of technological change in driving production increases. The aggregated effect of technology, or technical change, is measured by total factor productivity [3]. Total factor productivity is calculated as the ratio of output to an input index [4]. As such, total factor productivity growth is the residual growth in output that cannot be accounted for by growth in input use [11]. The higher the output obtained with a fixed set of inputs, the higher the total productivity of all the production factors, namely land, labour and capital^1^. Total factor productivity accounts for all inputs simultaneously, making it the appropriate metric to assess whether an agricultural system is becoming more efficient. Partial factor productivity measures such as yield, by contrast, credit a single input with gains that may actually come from using more of the others. Changes in total factor productivity affect the cost side of farmers’ balance sheets—producing the same output with fewer inputs is a reduction in the costs of production. Total factor productivity implies proportionality: a 1% increase in total factor productivity implies that all the land and non-land inputs became 1% more productive.

Beyond reducing individual farm costs, total factor productivity growth plays an important role in determining the trajectory of world commodity prices. By enabling the global food system to produce more output with fewer inputs, technological change has historically driven agricultural prices down relative to the average cost of other goods and services in the broader economy [12–14]. This long-term decline in real prices is critical for land sparing: as global productivity increases, the resulting drop in price reduces the economic incentive for farmers to clear marginal lands that have higher production costs [14,15]. This price-reduction effect ensures that the efficiency gains from innovation are passed through to consumers rather than solely being captured as profit and reinvested into further land conversion [12].

In practice, most technologies have a bias, as they are aimed at increasing the productivity of a specific production factor, say land or labour. The technologies of the Green Revolution, including high-yielding varieties, fertilizers, pesticides and irrigation, are innovations mainly targeted at increasing yields, so they are biased towards land savings. Multi-cropping is another example of a land-saving innovation. Other innovations such as animal draft and mechanization are labour-saving (and capital intensive).

Box 1:
**Economic concepts underlying rebound effects**

**Factors of production:** land, labour and capital. Factors provide a flow of services to production and are rewarded with factor payments: land earns rent, labour earns wages, and capital earns interest (or a rental rate). They are different from intermediate inputs such as fertilizer, seed and fuel, which are used up in production.

**Price taker:** a producer (or buyer) too small to influence the market price, who must accept the prevailing price as given. Because any one such producer supplies a negligible share of the market, they can sell as much as they want without moving the price, so the demand it faces is (nearly) perfectly elastic.

**Price elasticity of demand:** the percentage change in quantity demanded when prices change by 1%. Denoted as *e* < 0.

**Elastic demand:** consumers are highly responsive to price changes. A price reduction triggers a proportional (*e* = −1) or more than proportional (*e* < −1) increase in consumption.

**Inelastic demand (−1 < *e*** **< 0):** consumers are relatively unresponsive to price changes. The percentage drop in price exceeds the percentage increase in quantity demanded. Global food demand is typically inelastic owing to physiological limits on consumption (see §3.2 for discussion of non-food uses).

**Excess demand elasticity (*e*_ED_** **):** the demand elasticity faced by farmers in a region open to international trade, *e*_ED_ = [*e*_WD_ + (1−α)ϵ_RS_]/α, where *e*_WD_ is the global demand elasticity, α the region's share of global output and ϵ_RS_ is the supply response from competitors elsewhere. *e*_ED_ is more elastic the smaller the region: *e*_ED_ → ∞ (perfectly elastic) as α→0, and *e*_ED_ → *e*_WD_ as α→1. The (1−α)ϵ_RS_ term captures the international displacement effect: a larger foreign supply response ϵ_RS_ makes the excess demand more elastic [16].

**Intensification:** increasing agricultural output by using more inputs on existing farmland.

**Rebound effect:** market response that offsets the land-saving potential of a productivity improvement. Ranges from partial rebound (some land savings are lost to higher consumption) to backfire (see figure 2).

**Jevons effect (backfire rebound):** the extreme case of rebound, where productivity gains raise farming profitability enough to induce land expansion, so that total cultivated area increases. Named after economist William Stanley Jevons, who observed that improvements in steam engine efficiency paradoxically increased coal consumption [18].

**Total factor productivity (TFP):** the ratio of total agricultural output to a weighted index of all inputs. TFP growth measures the residual increase in output that cannot be explained by greater input use. It is the component of yield growth attributable to technological change rather than intensification [11,19].

**Technology spillovers:** the indirect land-use effects of productivity growth in one country on agricultural production and land use in other countries, transmitted through international commodity markets.

**Partial factor productivity:** output relative to a single input, such as output per worker (labour productivity) or per hectare (yield). Can rise merely by adding other inputs (e.g. fertilizer), whereas total factor productivity rises only when output grows faster than total input use.

De Janvry & Sadoulet [12] identify other innovations that are not necessarily tied to a production factor that are critical for improvements in factor productivity. Risk-reducing technologies focus on improving resilience against adverse supply shocks, and include crops tolerant to abiotic (heat, drought and flood) and biotic stresses (pest and diseases), as well as diversified farming systems. Quality-improving innovations enhance the nutritional value of crops (e.g. high-lysine corn), enhance shelf-life or allow market price premiums, such as organic agriculture. Finally, some innovations minimize environmental damage or promote environmental services in a way that reduces constraints in the use of resources. For example, innovations that reduce the need for fertilizers may lead to reduced water pollution [12,20].

Technologies and innovations aimed at one goal, increasing resilience and improving soils generally interact with other factors of production affecting their productivity. For instance, the introduction of herbicide-resistant genetically engineered seeds in Brazil allowed for no-tillage, thus reducing the need for ploughing and weeding, thereby reducing the amount of labour required per unit of land [21]. Just as reducing the amount of land required per unit of output increases the productivity of each plot of land, by reducing the number of workers in a given plot, labour-saving innovation increases the productivity of farm labourers.

It is often the case that increasing the efficiency of a factor, say making land more productive, increases the need for more intensive use of inputs. Prominently, the high-yielding varieties of the Green Revolution increased the need for labour and fertilizer [22,23]. If these inputs are not more productive in themselves, they represent a cost for the farmer.

This is why the distinction between technical change and intensification matters: technical change allows producing more with a fixed set of inputs, while intensification requires using more inputs to increase production. Yield growth in itself confounds innovation and intensification, and it is, therefore, a poor measure of technical change [24]. Changes in measured total factor productivity, on the other hand, captured the individual contribution of different technologies, taking into account their biases and the overall effects of the technology in the production process, into an overall index of input use efficiency [11].

Figure 1 decomposes average annual growth in agricultural production into four drivers: expansion of agricultural land, irrigation, intensification, and total factor productivity. The last three drivers are sources of yield growth [19]. Consistent with the evidence discussed above, figure 1 shows that between 1960 and 2010, crop-land expansion has been a relatively minor source of growth in production, explaining around 10% of annual growth in the period 1961–1990, and from 3% to 5% during 1991–2000. Looking at yield growth, there is a clear switch from growth in production based on input use, which explained over 80% of aggregate yield growth in 1961–1970, to greater reliance on productivity improvements, which supported around 70% of the growth in agricultural output in the period 1991–2010. The growth in productivity during this period was fuelled by the maturation of Green Revolution technologies, the rapid adoption of genetically modified crops and structural reforms in major developing economies, notably China and Brazil [15,21,26,27].

**Figure 1.**
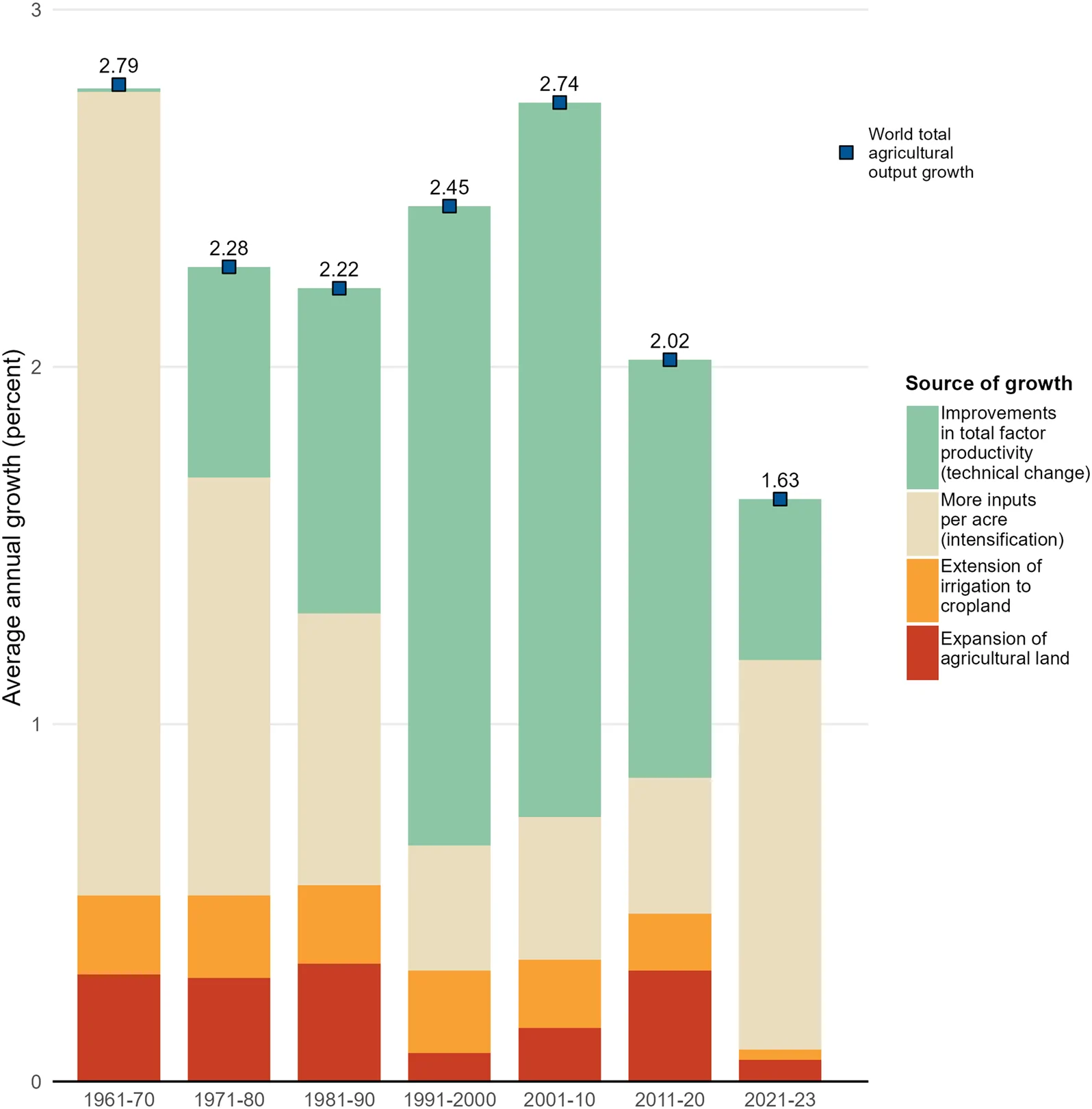
Sources of growth in global agricultural output, 1961–2022. Reproduced from USDA Economic Research Service, *International Agricultural Productivity* [25]. Data and methods as of January 2026.

Concerningly, the most recent periods (2011–2023) show a productivity slowdown, with global average annual total factor productivity growth declining sharply from 2.00% (during 2001–2010) to 1.17% in 2011–2020 and to 0.45% in 2021–2023 [19,25]. Improvements in total factor productivity explained close to 60% of the growth in agricultural output during the 2011–2020 period, and only 27% during 2021–2023. Furthermore, land expansion regained significance, with its contribution to output growth during 2011–2020 more than doubling compared with the preceding decade; by contrast, more inputs per acre explain two-thirds of agricultural output growth during 2021–2023.

The reduction of total factor productivity growth rates is attributed to several critical factors, including the long tail of underinvestment in public research and development, or R&D [28,29]. The decline in R&D funding, particularly in the USA, where public expenditures reached their lowest level since 1970 [20,26], hastened the erosion of accumulated knowledge, given the long-time lags between R&D investment and realized productivity gains [29,30]. This slowdown is also linked to the increasing drag of climate change, as global warming is estimated to have a negative and statistically significant impact on agricultural total factor productivity growth rates [31].

Another hurdle to further productivity growth is transferring technologies across different agro-ecologies [15]. Agricultural technology is inherently sensitive to location and requires local adaptation, which, combined with weak national research capacity, often results in less-effective innovation systems in developing countries [15]. Moscona & Sastry [32] find that the ecological mismatch between the agro-ecological environments of the global leaders in agricultural R&D and those of potential recipient countries explains, on average, around 20% of cross-country agricultural productivity differences. The countries most ecologically distant from the innovation frontier, especially those in sub-Saharan Africa, suffer the largest productivity penalties, making locally adapted agricultural R&D particularly critical there.

The impact of this productivity slowdown is far from uniform, revealing a widening gap between regional leaders and countries that are falling behind. South Asia has emerged as a global leader in agricultural productivity growth, with India successfully transitioning from resource-intensive growth toward innovation-driven productivity gains [33]. By contrast, sub-Saharan Africa continues to lag as the clearing of new lands for cultivation still accounts for at least two-thirds of all gains in agricultural output [33]. This regional failure to improve R&D efficiency and manage climatic shocks means that output gains in the most vulnerable regions continue to come at the direct expense of natural habitats [15,20].

A natural concern is whether the slowdown in agricultural total factor productivity owes to the possibility that the global leaders in agricultural R&D have exhausted the biological potential for yield growth^2^. Recent evidence on this is provided by Gerber *et al.*, who use worldwide data on spatially explicit yields for the period 1975–2010 for 10 crops comprising over 80% of global caloric consumption to estimate attainable yields^3^ in eight large regions of the world and spatially explicit yield gaps, defined as the difference between grid cell level average yields and regional attainable yields. They classify each pixel into one of three typologies of yield gap closure: ‘steady growth’, whereby both attainable yields and actual yields are growing, the latter at a slower pace than the former, so yield gaps widen over time; ‘stalled floor’ where attainable yields are growing but yield gaps are increasing because of stagnated actual yields; and ‘ceiling pressure’, in which yield gaps are closing because actual yields are converging to stalling attainable yields.

They find that for maize, 60% of global harvested maize area is experiencing steady growth, while 25% faces ceiling pressure, and that all the different outcomes coexist in most regions. In the case of soybeans, 48% of the global harvested soybean area is experiencing steady growth, while 41% is facing ceiling pressure, and the rest falls into the stalled floor category—again, with all producing regions displaying heterogeneous outcomes. This heterogeneity within leading R&D regions seems inconsistent with an exhaustion of their biological yield potential. Wheat is the clearest case of ceiling pressure in a high-income region, north-west Europe, a fact documented by Grassini *et al.* [34], and which recent research attributes to agronomic management [35] and to environmental policies that require reduced inputs such as fertilizers [35–37], rather than to limitations owing to genetics or climate. Meanwhile, North American wheat spans the full range of outcomes. In the case of rice, in much of the tropics, and particularly in major Asian producers, actual yields are rapidly converging on attainable yields, so further gains depend on research that raises both attainable and actual yields [37].

Yield trends, however, are an incomplete measure of productivity. Yields relate output to land alone, whereas total factor productivity relates output to all inputs jointly, and the two can diverge sharply. According to Hertel *et al.* [14], between 1990 and 2016, the aggregate amount of land, labour, capital and intermediate inputs used in north-west European agriculture declined by about one-fourth owing to reforms to EU agricultural policy. Yet farm output did not drop owing to continued improvements in total factor productivity. In the USA and Canada, by contrast, aggregate inputs remained almost constant over 1990–2014 and steady growth in total factor productivity translated into rising output. In other words, the same productivity growth was directed toward reducing input use and environmental damage in Europe, and towards expanding food supply in North America [14]. A yield plateau is therefore consistent with continued technological progress, highlighting the differences between factor productivity and yield growth and emphasizing that measuring TFP growth requires measuring inputs and outputs jointly rather than reading yield trends alone.

The evidence discussed above leaves little room to question, at least at the global level, the potential of technology-driven yield growth to save land. Why then are we worried about higher yields not sparing land for nature? The reason is the existence of rebound effects—behavioural and market responses that can diminish land savings—and especially, their extreme form, the Jevons effect, which can turn land-saving potential into further expansion.

## 3. When do yield increases lead to land expansion?

Examples of technology-driven land expansion are commonly found in the production of commercial commodities near agricultural frontiers. For instance, the introduction of herbicide-resistant soybean seeds in Brazil significantly reduced labour costs by allowing for no-tillage farming. This increased profitability incentivized farmers to expand soybean production into sensitive biomes like the Amazon and Cerrado, often displacing existing cattle pasture and pushing ranching further into the forest frontier [1,22]. A similar process has been documented in the Chaco region of Argentina [38]. The introduction of irrigation in the lowlands of Palawan, the Philippines, drew labour away from the forest, but the resulting surge in household wealth eventually allowed farmers to reinvest in upland expansion, clearing more forest to plant high-value crops on the hillsides, reversing early land-saving gains [39,40]. A similar pattern of upland deforestation following the introduction of improved seeds and fertilizers in lowland rice production in central Indonesia is documented by Maertens *et al.* [41].

Recent work by Jeddi & DePaula [42] uses twice-lagged second-season weather shocks combined with soil clay content as instrumental variables (or exogenous shifters) of the profitability of double cropping to identify the effects of increases in double cropping shares on crop-land expansion across Brazil. They find that in Brazil's agricultural frontier, where land is abundant and relatively cheap to convert, the adoption of double cropping that accelerated in the early 2000s is responsible for 44% of new crop-land expansion (5.5 Mha) in the region. By contrast, in consolidated agricultural areas of Brazil, double cropping has no significant effect on crop-land expansion. Similar findings are reported by Richards & Arima [43], who find that deforestation in the Brazilian state of Mato Grosso is driven by surges in sector-level profits following the introduction of soybean-corn multi-cropping, which increase the availability of investment capital, drive up land prices and incentivize speculative clearing. Further evidence on the effects of agricultural technical progress on crop-land expansion is provided by Akerman *et al.* [44], who find that the staggered establishment of research centres by Brazil's Embrapa across different ecological zones caused significant yield growth for targeted staple and commercial crops (such as soybeans and maize), which in turn drove an expansion of total crop-land.

The dichotomy between the technology-driven global land savings documented at the beginning of this essay and the technology-driven land expansion in agricultural frontiers underlies the enduring debate between the ‘Borlaug hypothesis’, which posits that higher yields spare natural habitats by reducing the land required to meet food demand, and the ‘Jevons paradox’, which suggests that those same efficiency gains increase the profitability of farming, thereby incentivizing the conversion of forests into crop-land [24].

### 3.1. Demand responsiveness or elasticity

All the examples above, and all the instances where technological change results in land expansion, have one element in common—the regions where the innovation takes place face an elastic demand for their products [16,22].

Conversely, work focusing on the introduction of technologies on staples or subsistence agriculture generally finds a reduction in land clearing [45–49].

Technically, the elasticity of demand measures the percentage change in consumption following a 1% change in prices. This parameter determines how productivity gains are distributed between consumers (through lower prices) and farmers (through higher returns to land)—and thus whether farmers face incentives to expand or contract their operations. Figure 2 illustrates this mechanism, starting with the definition of rebound effects in terms of yield-area relationships (figure 2a, adapted from Balmford [17]) and then showing the underlying economics (figure 2b,c).

**Figure 2.**
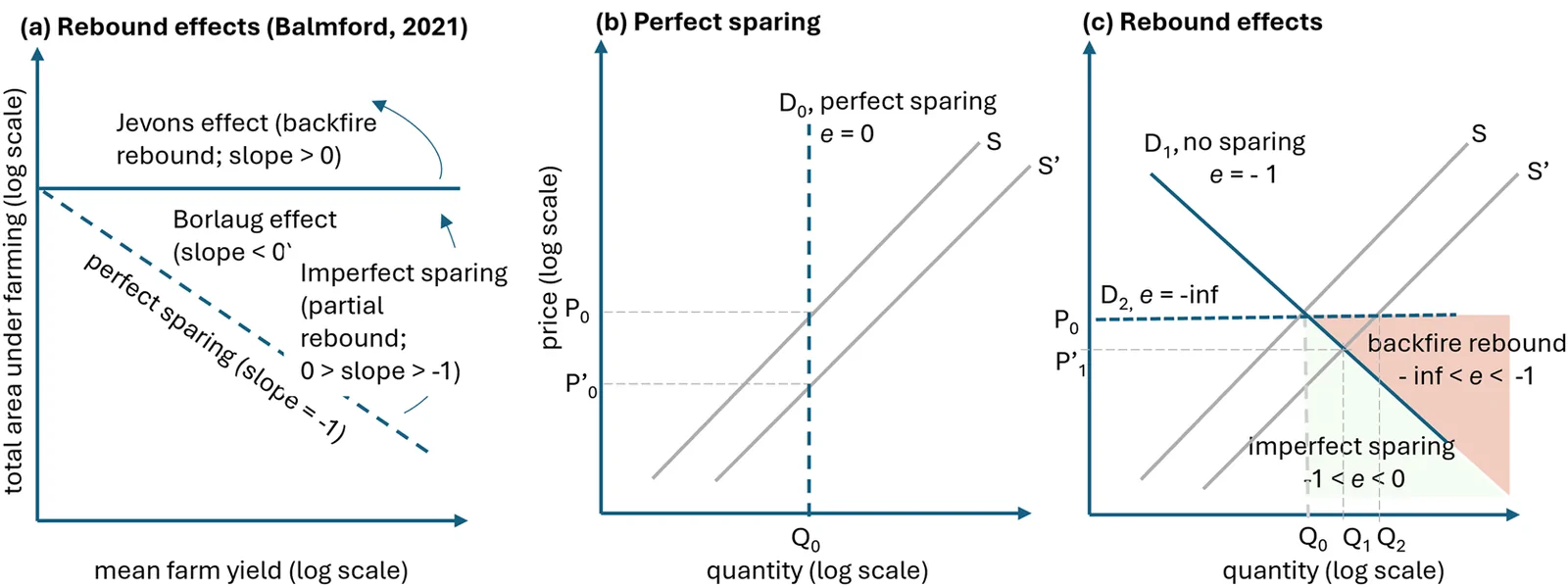
Technical change, demand elasticity and rebound effects. (a) Rebound effects (adapted from [17]). Balmford [17] shows the relationship between mean farm yield and total area under farming (both in log scale). Perfect sparing corresponds to a slope of –1; imperfect sparing (partial rebound) to slopes between –1 and 0; no net land change to a slope of 0 and backfire to positive slopes. (b) Perfect sparing is an engineering benchmark that assumes perfectly inelastic demand (*e* = 0). Productivity improvement shifts supply from S to S′, but quantity demanded remains fixed at Q₀. Prices fall from P₀ to P′₀ by the full extent of the productivity gain—cost savings are entirely passed through to consumers, leaving returns to land unchanged. Land requirements fall in direct proportion to the productivity increase. (c) Rebound effects are determined by the elasticity of demand faced by the producers experiencing technical progress. When demand responds to prices (*e* < 0), the lower prices resulting from the shift from S to S′ result in greater consumption and therefore markets clear at quantities that exceed the benchmark Q₀, partially or fully offsetting potential land savings. With inelastic demand (–1 < *e* < 0, green region), prices fall more than costs, squeezing returns to land; production rises to Q₁, but land is still spared (imperfect sparing or partial rebound). With elastic demand (–∞ < *e* < –1, pink region), prices fall less than costs because consumers readily absorb additional production; returns to land rise, production expands to Q₂ and land use increases—termed backfire or Jevons effect. The boundary case (*e* = –1) results in no net land change (100% rebound).

Figure 2b shows the benchmark case of perfectly inelastic demand (elasticity = 0), where consumers purchase a fixed quantity regardless of price. By definition, improvements in total factor productivity shift the supply schedule from S to S′ (intuitively, quantities can be produced at lower prices). With quantity Q₀ fixed, prices fall by the full extent of the productivity gain—from P₀ to P′₀. The entire benefit of innovation is passed through to consumers as lower food prices. For farmers, production costs fall but so do the prices they receive, leaving returns to land unchanged; therefore, land requirements decline in direct proportion to the productivity increase—this is perfect sparing, corresponding to a slope of –1 in figure 2a.

Figure 2c shows what happens when demand responds to price changes. Consider first the case of inelastic demand, −1 < *e* < 0, which would characterize any demand schedule (line) in the imperfect-sparing zone in light green. As in figure 2b, improvements in total factor productivity shift the supply schedule from S to S′. When demand is not perfectly inelastic, the lower prices from increased supply induce some additional consumption (from Q₀ to a value in between Q₀ and Q₁ in the figure), but the percentage increase in quantity demanded is smaller than the percentage drop in price. To illustrate, assume that the elasticity of demand *e* = −0.2 and that total factor productivity increases by 10%, which implies a proportional 10% reduction in unit production costs, including the amount of land needed to produce a given quantity. Further assume that the increase in production (from Q₀ to Q₁) needed to satisfy demand at the new lower price is 2.5%; by the definition of elasticity, this implies a reduction in prices of 12.5%. In other words, when demand is inelastic—its absolute value is less than one—the percentage drop in prices needed to clear the market exceeds the percentage reduction in production costs, so returns to land shrink relative to the scenario without the technology. This is the mechanism that ensures technology-driven yield growth remains land-sparing: while some land savings are ‘lost’ to higher consumption, the overall area under cultivation still contracts because the economic incentive to expand has been removed. This is partial rebound, corresponding to slopes between −1 and 0 in figure 2a.

Now consider elastic demand, –∞ < *e* < –1, which describes all the demand schedules in the backfire rebound region in light red. When demand is elastic, a small price reduction triggers a large increase in consumption. Prices need only fall modestly to clear the additional production, so the price decline is smaller than the cost reduction from productivity growth. As shown in figure 2c, if demand is perfectly elastic (*e* = –∞), farmers would expand production to Q₂ without a change in prices. Because prices decrease at a slower rate than the reductions in costs, farmers′ profit margins expand. With higher returns to land, it pays to bring additional or marginal lands into production. Farmers respond by expanding their operations and increasing total land use. This is the so-called backfire rebound effect, corresponding to positive slopes in figure 2a. The boundary case of unit elastic demand (*e* = –1), shown as D, marks the threshold—the increase in production exactly offsets the productivity gain, leaving land use constant. So, the unit elastic demand acts as a switch: any demand line steeper than this (in the light green zone) results in a price drop that outstrips cost savings, leading to sparing. Any line flatter than this (the light red zone) means cost savings outstrip the price drop, triggering a Jevons effect, provided that there is land available for crop-land expansion.

### 3.2. Geographic scale and commodity tradability

The elasticity of demand is in itself a function of the tradability of the product under consideration and the geographic scale of the market. For a farmer in an isolated village without ready access to markets or producing non-tradable, perishable staples and produce, adopting technologies that increase output would quickly drive prices down, reducing farm profitability, and thus the incentives to expand land. Conversely, from the perspective of a single farmer in Brazil's Mato Grosso, or in the USA Midwest, the demand for soybean, a highly tradable commodity, is perfectly elastic—these farmers can always place an extra bean in global markets and the prices they receive will not budge—every additional soybean brings additional revenue, and because the additional production is the result of better technology, revenues grow faster than production costs increasing farming profitability and with it the incentives for land expansion. But as we move towards a larger scale, aggregated increases in production are not innocuous anymore; a bumper crop in Brazil (or the USA) will most likely push global prices of that crop down. This is because at more aggregate levels, demand loses elasticity; the larger the market share of a country or region, the more responsive prices are to changes in quantities.

When we take this logic to a global context, the aggregated market share of agricultural products of all the farmers in the world is 100%; therefore, any change in quantities will be accompanied by large, more than proportional changes in prices. In other words, globally, the demand for food is inelastic [50]. We live in a closed global system where, in the short to medium run, income and population are relatively fixed: with physical and physiological limits to consumption, a drop in global prices does not lead to a proportional increase in total consumption. At the global level, we face the same experience of the farmers in an isolated village or producing non-tradable staples to which I alluded above.

Direct human consumption is only one use of agricultural output. West *et al.* [51] estimate that in 2020, 45% of global crop calories were consumed directly as food (including processed foods), 40% were used as animal feed, 5% were used to produce biofuels and the rest were destined for industrial uses. The price elasticity that producers face at the farm level is best understood as a sales-share-weighted sum of the demand elasticities arising from each of these uses [52]. As mentioned above, physiological limits to consumption result in inelastic demand for agricultural output destined for human consumption—limits to consumption typically apply to both processed foods and livestock products. More generally, the effects of an increase in the share of agricultural products destined either for processed food or for livestock feed on the farm-level elasticity depend on the degree to which the raw agricultural inputs are substitutable and the price demand elasticity of the final product.

When substitution possibilities are limited, the derived demand for feed at the farm level tends to be more inelastic than the demand for the corresponding product at retail [53]. Available estimates indicate that the demand for livestock products is inelastic [54,55] and the global elasticity of demand for wheat, corn and soybeans, for both feed and food, is highly inelastic^4^ [50,57]. Income reinforces the low responsiveness of farm output to changes in prices. Consumers in poor countries devote larger shares of their disposable income to food and are correspondingly more sensitive to changes in prices, while individuals in wealthier nations spend less on food and are less prone to adjust their consumption patterns because of changes in prices, so that income growth tends to render food demand progressively more inelastic [54,55].

The effects of increased biofuel production on the farm-level demand for feedstocks depend on specific biofuel policies. Biofuel mandates require fixed quantities of feedstocks regardless of prices, so the corresponding demand for feedstocks for biofuels is perfectly inelastic for binding mandates; this reduces the absolute value of the farm-level elasticity for feedstocks [56]. However, when oil prices are high enough to stimulate production beyond the mandated volume, feedstock demand becomes more price responsive [58]. As with feedstocks, the effects on the farm-level demand elasticity of novel sources of demand such as cosmetics will depend on the price responsiveness of the final good and on the substitutability of the farm product within it. In sum, as non-food uses, particularly those where agricultural commodities are more easily substituted by alternative inputs, account for a growing share of total demand for farm products, the farm-level elasticity of demand may become less inelastic, weakening the land-sparing potential of broad-based yield growth.

Table 1 summarizes the effects of technological progress on land expansion depending on whether the relevant demand elasticity is elastic, which in turn depends on the scale of analysis and on whether the products are tradable. Just as the global demand elasticity for farm products could rise with the growth of non-food uses, the boundaries between rows of table 1 are not fixed: places currently isolated could become integrated with the improvement of transportation networks [59], raising the demand elasticity they face and amplifying rebound effects. Asher *et al.* [60] document this mechanism in India, where they find that construction of last-mile rural feeder roads had no effect on forest cover, while upgrades to national highway corridors connecting production to larger markets caused a significant decline in nearby forest cover. Gollin & Wolfersberger [61] quantify the combined role of market access and technical change in Brazil, estimating that transport cost reductions since the 1990s account for nearly a 10th of total deforestation, and that when road improvements coincided with the adoption of new soybean varieties, the joint effect rose to 40% of observed forest loss.

**Table 1.** Conditions under which technical change in agriculture leads to land expansion.

scale/context	excess demand characteristic	economic result of productivity growth	land use consequence
global	inelastic: limited by population and satiety^5^	prices fall significantly	land sparing (Borlaug)
local (isolated)	inelastic: limited by local population (non-tradable)	local prices crash; farm revenue may drop	land sparing
local (integrated)	elastic: producers can export to the world (tradable)	producers capture market share; profits rise	land expansion (Jevons)

### 3.3. International trade

In addition to the scale of analysis, international trade also plays a critical role in the land use effects of technological progress. In essence, when a country experiences the productivity boost of an innovation, it reduces the need to produce in other parts of the world. For instance, increases in the productivity of export crops in the USA lead to land expansion within the USA owing to rebound effects [62]. However, as the USA displaces Brazilian production from global markets, innovations in the USA offset the pressures to clear land in Brazil^6^. These market-mediated effects turn out to be quite important, as we will discuss next.

### 3.4. Direct and indirect land use effects of agricultural productivity growth

In [62], I estimated the effects of growth in domestic total factor productivity on domestic crop-land expansion, foreign crop-land expansion and global crop-land expansion for a sample of 70 countries^7^. Following FAO, crop-land is defined as the sum of arable land and permanent crops; pastures are excluded. The estimating equation corrects for cross-country and temporal changes in demand, land rents and input prices. The underlying theory conditions the effects of growth in total factor productivity on openness to international trade and competition with other countries and derives expressions for the effect of domestic growth in total factor productivity on domestic crop-land, on foreign crop-land and on global crop-land. The effects of domestic growth in total factor productivity on domestic land use measure the local rebound effects, the effects on foreign demand measure the importance of market-mediated effects and the effects on global crop-land take into account the offsetting effects of land savings abroad on domestic rebound effects.

The domestic crop-land elasticities, defined as the percentage change in domestic crop-land when domestic total factor productivity increases by 1%, are displayed in the first row of figure 3. The elasticities displayed are based on OLS estimates, which are the preferred specification in [62]^8^. The error bars represent the uncertainty in the underlying regression estimates. The most striking pattern is that only in very few countries—India, China, Nigeria, Pakistan, Turkey and Bangladesh—the point estimates of the crop-land elasticities are negative. That is, given the exposure to international trade in 2001–2010, very few countries show evidence that increases in domestic total factor productivity result in domestic land sparing. For the vast majority of the 70 countries in the sample, the effect is numerically indistinguishable from zero, or points towards rebound effects. The countries with the strongest rebound effects—Canada, the USA, Argentina, Brazil, European countries and commodity exporters in sub-Saharan Africa—are all commodity exporters highly integrated into world markets^9^. Another conclusion of this work is that the pervasiveness of the Jevons effect is likely to increase as countries become more engaged in international trade. The underlying mechanism is that the more integrated into the world economy a country becomes, the higher the elasticity of demand faced by its farmers becomes. This shift is visible in figure 3, where several countries in Africa, South America and Asia display larger positive domestic crop-land elasticities in 2001–2010 than in 1991–2000, reflecting their deeper integration into world markets over this period.

**Figure 3.**
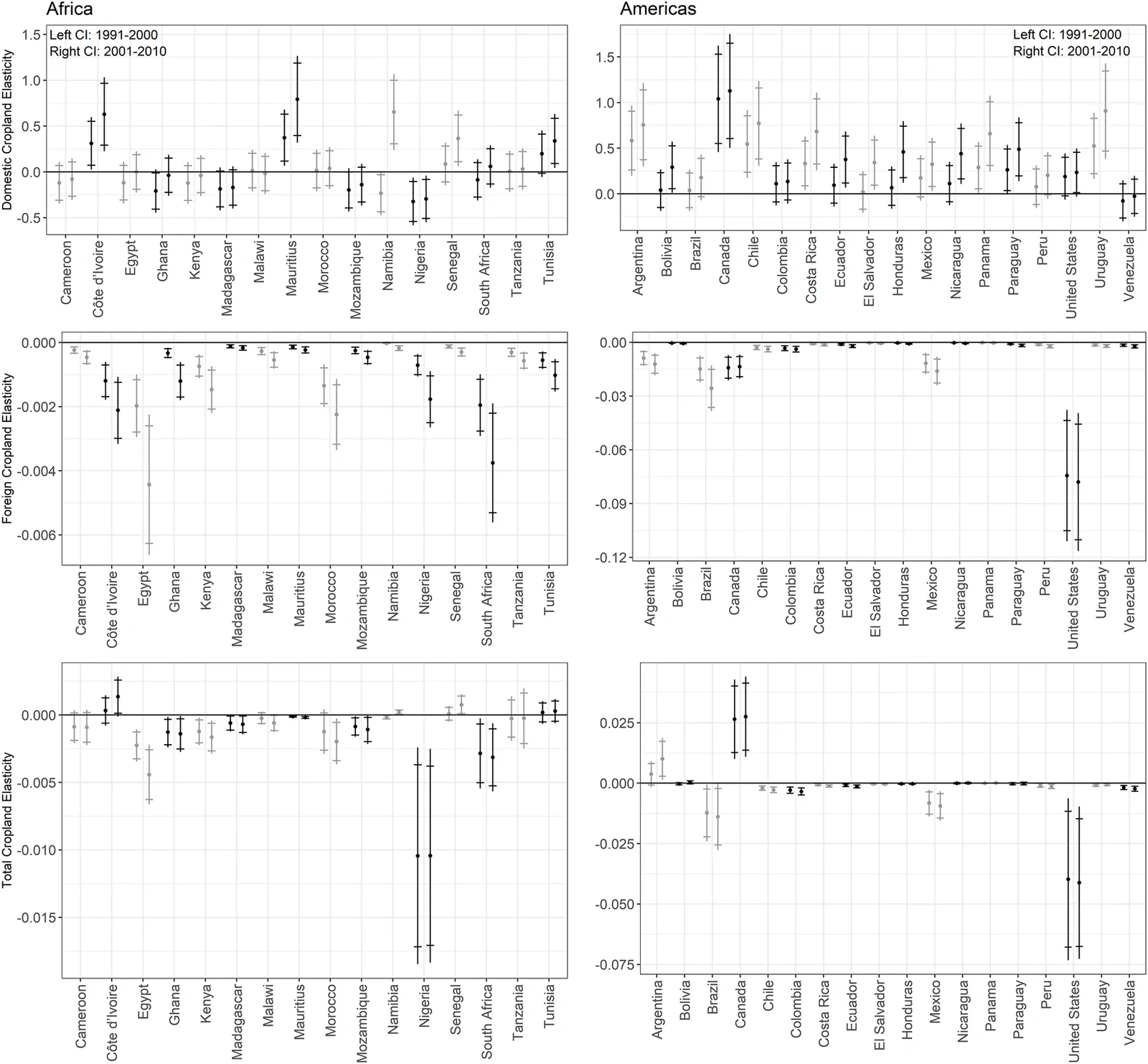

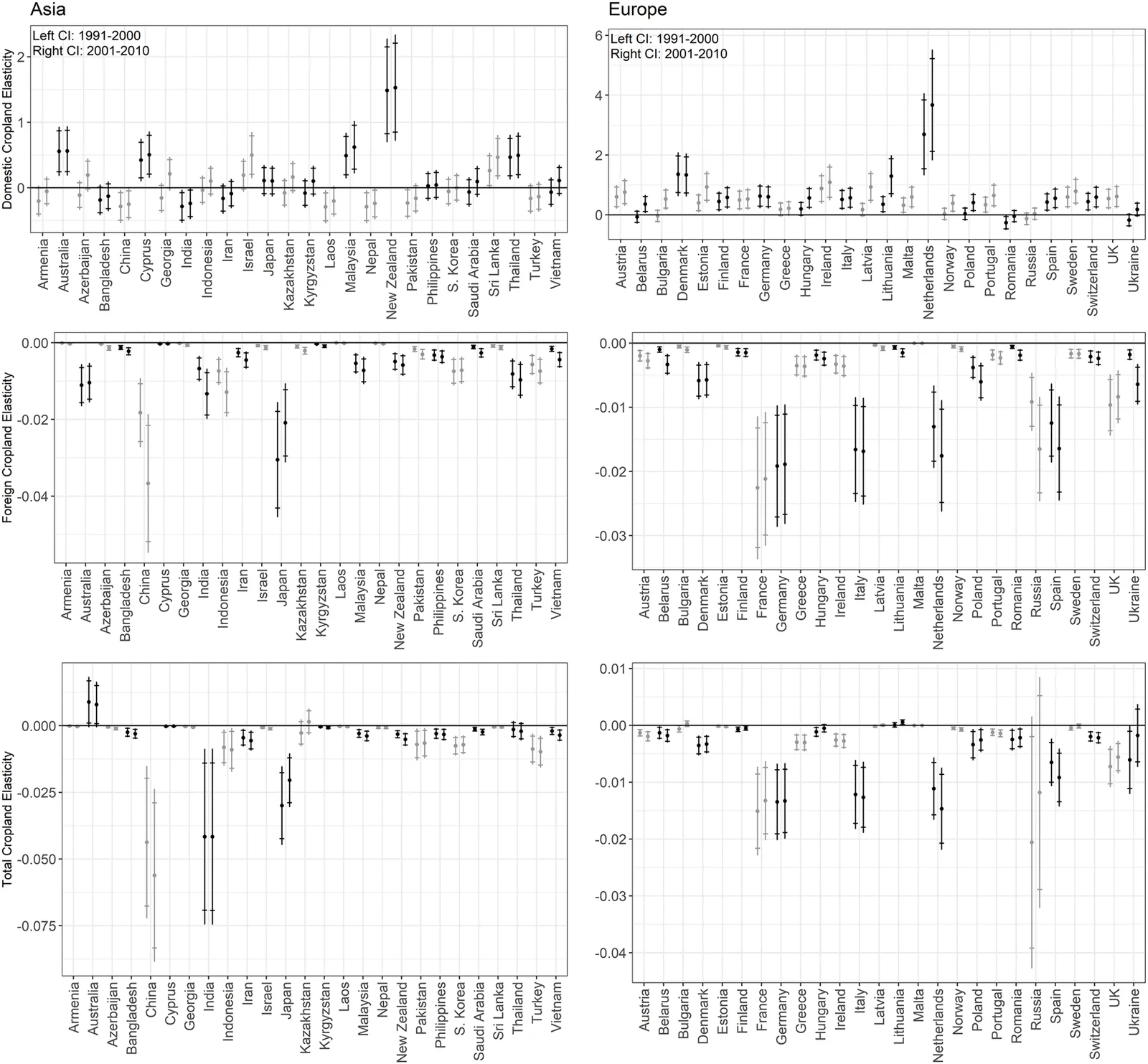
Domestic, foreign and global crop-land elasticities with respect to total factor productivity (TFP). The figure displays the median point estimates (dots) and confidence intervals (90% identified by horizontal tick marks, 95% by the interval extent) for the elasticity of crop-land area with respect to TFP for 70 countries. Each panel reports estimates for a different region. Black and grey error bars alternate between adjacent countries to aid visual separation. The top, middle and bottom rows show the domestic, foreign and total crop-land elasticities, respectively. The elasticities are estimated based on crop-land, trade and TFP data for 2001–2010, following the econometric framework established in [62]. Reproduced from [62]. Licensed under CC BY-NC 4.0.

Domestic responses, however, are an incomplete answer to the question of whether technological progress spares land for nature. The second row of figure 3 shows the effects of domestic growth in total factor productivity on land use abroad. This is a measure, other things being equal, of the market-mediated effects discussed above. By definition, these foreign crop-land elasticities are negative. Using Canada as an example, the upper row of figure 3 shows that doubling Canada's total factor productivity increases Canada's crop-land by around 30%. However, as Canada's production increases, it exports more, displacing producers elsewhere, so a doubling of Canada's agricultural total factor productivity reduces foreign crop-land by around 1% (second row of figure 3).

The last row of figure 3 shows the global effect of domestic innovations. This is a weighted average of the domestic and foreign effects. In the case of Canada, the Jevons effect overwhelms the land savings abroad: doubling Canada's agricultural total factor productivity leads to an increase in global crop-land of close to 2%. In this regard, Canada turns out to be an anomaly—except for Canada, Argentina, Australia and Côte d'Ivoire—domestic growth in total factor productivity leads to global crop-land savings, even if domestically we observe rebound effects.

Figure 4 offers additional insights on the diffusion of the effects of technology on land use across regions. Each panel shows an innovating region, identified by the orange bar at the top. The region's observed growth in agricultural total factor productivity during 2001–2010 is reported at the top right of each panel. The orange bars are the direct land use effects of productivity growth, that is, the change in regional crop-land as a response to regional changes in agricultural total factor productivity. The yellow bars are the indirect land use (i.e. extra-regional) effects of agricultural productivity growth in the innovating region, and the blue bars are the net global effect. The displayed changes in crop-land assume that other regions did not experience productivity growth, so the displayed changes correspond to the direct, indirect and global land use effects of region-specific changes in agricultural total factor productivity, all other things being equal.

**Figure 4.**
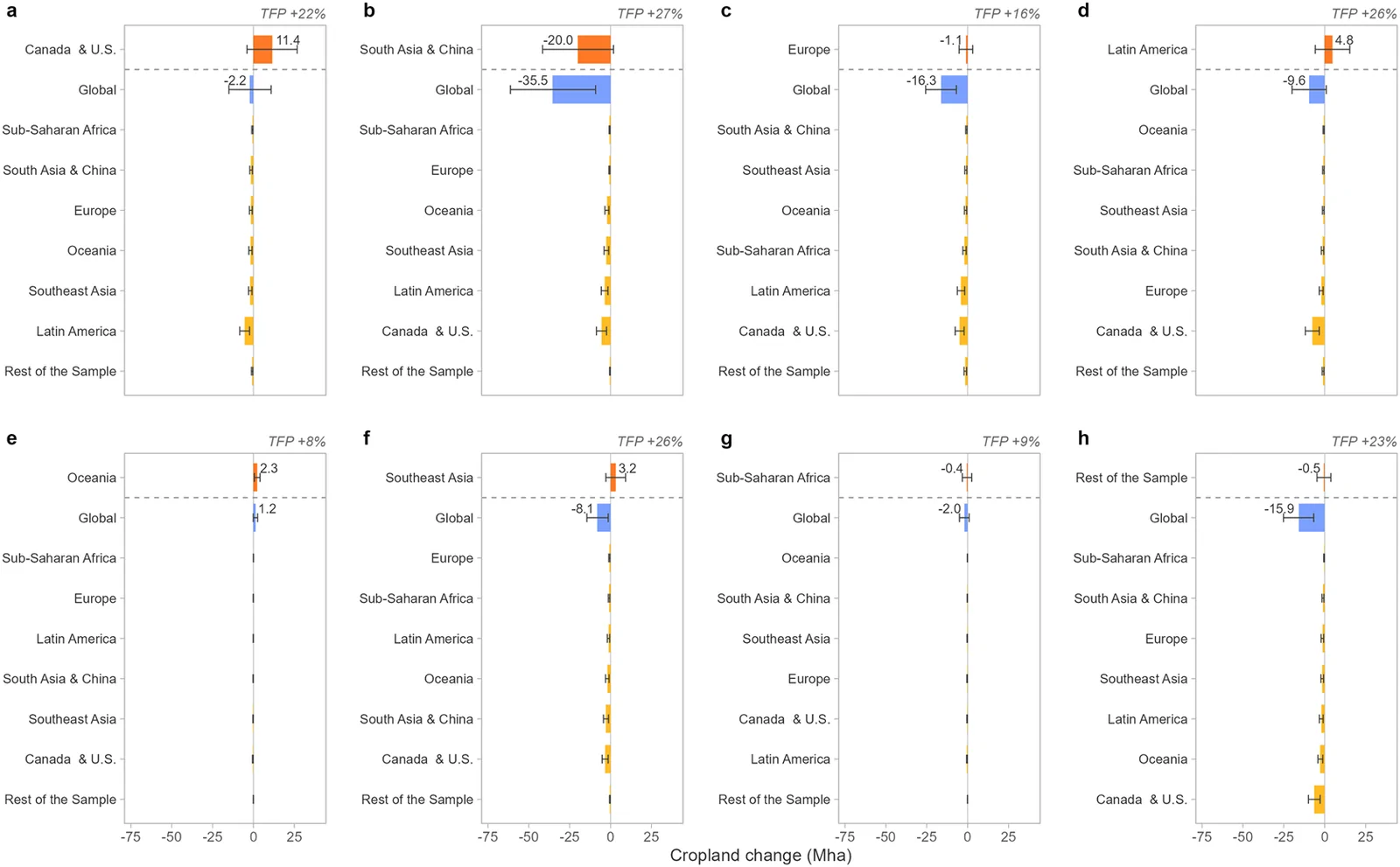
Direct and indirect land use effects of agricultural productivity growth. The panels show the estimated changes in crop-land (horizontal bars) across different global regions resulting from the level of TFP growth observed during 2001–2010, occurring only within the focal region, shown as the orange bar at the top of each panel. The observed TFP growth for that region during 2001–2010 is given at the top right of each panel. For example, (a) illustrates how a 22% increase in TFP in Canada and the USA (and assuming no TFP growth in other regions) drives land use changes globally, including significant contractions in other regions. Within each panel, a dashed line separates the focal region at the top from the global aggregate, the remaining regions and the rest of the sample at the bottom. Blue bars represent the aggregate net change in global crop-land. Labels are the point estimates of own and global effects. Adapted from [63]. Licensed under CC BY 4.0.

As before, the error bars represent the uncertainty in the underlying regression estimates from the effects derived in [62]. To illustrate, the observed 22% increase in productivity in North America during 2001–2010 was associated with a point estimate of an additional 11.4 Mha of crop-land within North America. Elsewhere, however, productivity growth in the USA would have reduced crop-land by around 2 Mha. Most of this effect operates by reducing crop-land in South America (by 5 Mha), as the USA displaced its South American competitors in world markets.

Notice that the only regions in the world where regional increases in total factor productivity reduce crop-land expansion significantly are in South Asia and China. Sub-Saharan Africa also displays crop-land savings, but at a lower scale owing in part to the limited growth in productivity experienced during 2001–2010.

### 3.5. Counterfactual: what would have happened without technological progress?

The discussion above focused on understanding the geographic heterogeneity of the effects of agricultural productivity growth on domestic, foreign and global crop-land. When we add up all the country-level new hectares of crop-land cleared to satisfy the strong demand for agricultural commodities on the one hand, and the hectares saved from being converted because prices were lower than in a counterfactual world without technological progress, the net result is the global land savings, documented in the introduction of this essay. In [62], I demonstrate that the global elasticity of crop-land with respect to changes in agricultural total factor productivity is a crop-land-weighted average of the domestic and foreign crop-land elasticities discussed above. The estimate for this global elasticity is −0.34, meaning that a 1% increase in global agricultural total factor productivity leads to a 0.34% reduction in global crop-land. Therefore, taking into account the scale of analysis reconciles the pervasiveness of the Jevons effect with the land-sparing effects of technology.

In [62], I demonstrate the way in which these effects build up by conducting a simple thought experiment: what would have been the crop-land needed in 1991–2010 in the absence of agricultural total factor productivity growth. Technically, these are regression predictions, and therefore the counterfactual assumes that demand, input prices and other unobserved factors affecting crop-land expansion remain constant. I estimate that in the absence of agricultural productivity growth, global agriculture would have required an additional 173 Mha of crop-land to satisfy the growth in demand driven by population and income. This contrasts sharply with the observed data for that decade, where the sample countries (representing approx. 75% of global crop-land) actually experienced a net contraction of approximately 2 Mha. By shutting down the land-saving effects of agricultural productivity growth, direct (including rebounding) and indirect (via trade), no region would have seen land contraction; instead, every region would have been forced to expand its agricultural footprint. For example, Latin America would have required an expansion of roughly 49 Mha, more than double the approximately 23 Mha actually observed.

In [63], I extend the analysis by mapping the country-level results discussed above onto a spatially explicit framework to evaluate the specific impacts of productivity growth on global biodiversity. This process involves apportioning national crop-land changes to 827 global ecoregions by assuming that crop-land expands or contracts within each ecoregion of a country at the same uniform rate calculated for that nation as a whole. By merging these econometric predictions with global datasets on crop-land extent and species richness for birds, amphibians and mammals, I further aggregate the data into 14 global biomes. The results, reproduced in figure 5 , show that in the absence of TFP growth, more than one-half of the additional land required—approximately 73 Mha —would have been cleared from the world's four most biodiverse biomes.

**Figure 5.**
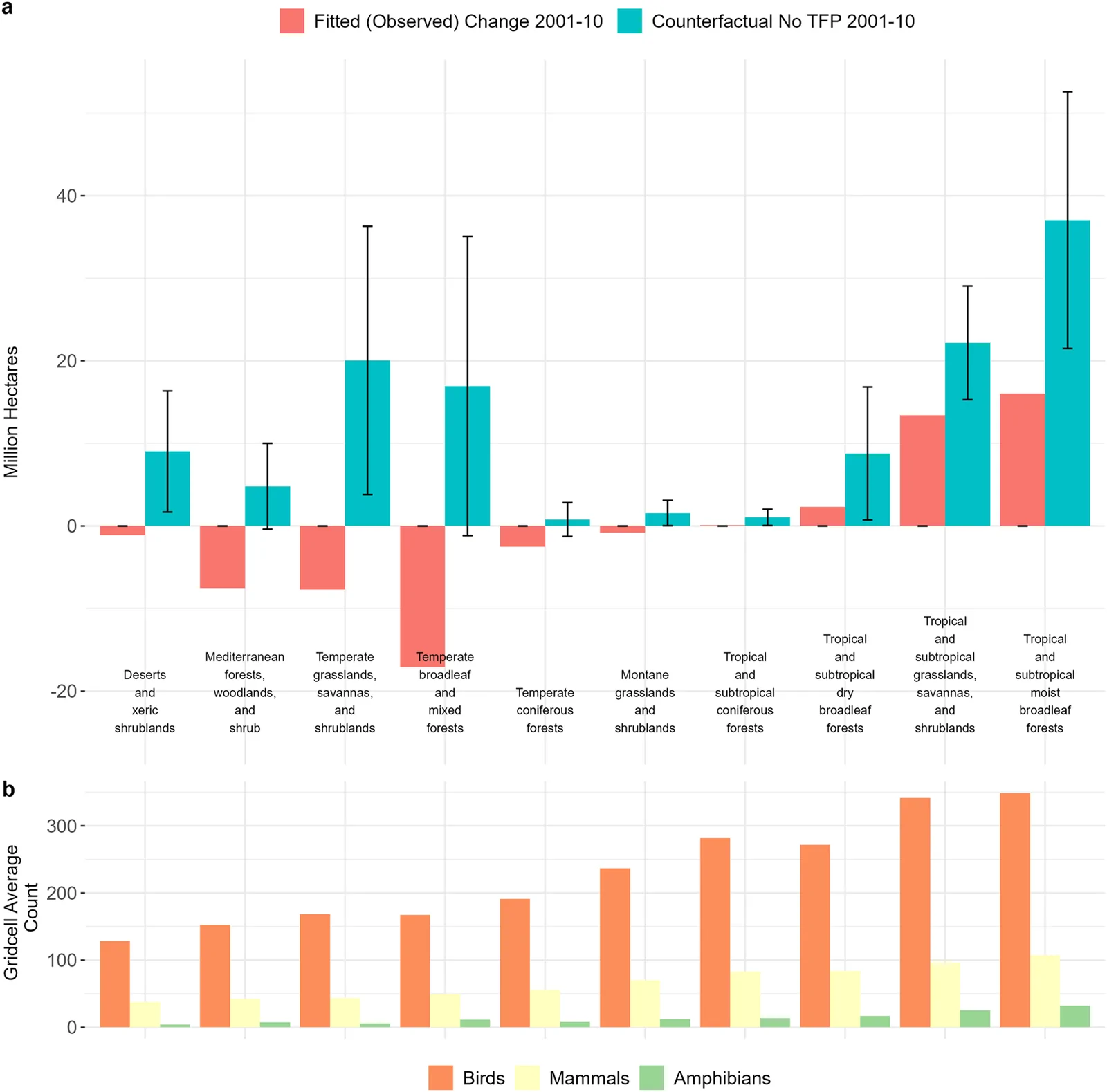
Counterfactual land needs by selected biomes. (a) The blue bars represent a counterfactual scenario, showing that without TFP growth (2001–2010), global agriculture would have required significant expansion into new lands. This expansion is most pronounced in the tropical and subtropical moist broadleaf forests. (b) Species richness (birds, mammals and amphibians) for each biome. Reproduced from [63]. Licensed under CC BY 3.0.

### 3.6. Can technical progress in agriculture result in global crop-land expansion?

The discussion above clarifies that the seeming contradiction between the Borlaug hypothesis on the one hand and Jevons paradox on the other hand can be explained by considering the scale of the analysis and the tradability of commodities under study. The documented cases of Jevons paradox occur in agricultural frontiers producing for markets with high demand, while the historical reliance of food production on yield growth using a relatively constant land base is consistent with Borlaug's hypothesis that yield growth spares land for nature.

This is not to say that technical progress focused on one region could not lead to increases in global crop-land use. Hertel *et al.* [16] show that for a region that is integrated into the global market, and thus faces a highly elastic excess demand, export expansion could overwhelm the land-sparing contraction in the rest of the world if two conditions are met. First, the innovating region possesses relatively low yields compared with global competitors. Second, the region has large amounts of cheaply accessible natural lands, which would make land supply highly responsive. The intuition is that to increase exports, the region could clear large amounts of low-yielding land as it displaces production from relatively high-yielding land elsewhere.

Hertel *et al.* [16] conclude that the historical Green Revolution in Asia and Latin America was unambiguously land-sparing because trade barriers kept markets segmented, resulting in farmers facing inelastic excess demands. They also argue that, conditional on current levels of trade, a prospective African Green Revolution poses a risk of initially increasing global crop-land use because it has yields that are significantly lower than the global average as well as a high potential for land expansion. They also show that if productivity growth is sustained for roughly 20 years, African yields will eventually converge to those of other regions, restoring a global land-sparing trajectory.

### 3.7. Factor-biased technologies

Regardless of whether we are concerned with the rebound effects of technical change in staples in settings disconnected from markets, or the backfire rebound or Jevons effect that arise in settings where farmers face an elastic excess demand, the bias of technical change has an important effect on determining the magnitude of rebound effects [22]; in general, rebound effects will be stronger when the new technology reduces the constraints on the most scarce factor of production at the agricultural frontier [22].

An example in the case of regions facing an elastic excess demand is the introduction of genetically engineered, herbicide-tolerant soybeans in Brazil, to which I alluded before. By facilitating no-tillage farming and reducing the need for manual weeding, this technology significantly lowered labour requirements per hectare. As it is often the case in agricultural frontiers, agricultural expansion in Brazil's Cerrado and Amazon biome is limited by the availability of labour. Thus, the introduction of new soybean seeds removed the primary constraint on farm size, incentivizing producers to clear and manage much larger areas of the Cerrado and Amazon biomes than was previously possible [21,64]. A similar labour-saving rebound driven by mechanized zero-tillage has been documented in the Argentine Chaco [38].

Another example is provided by Maertens *et al.* [41] and Shively & Pagiola [40], who studied deforestation dynamics in the upland settings of Indonesia and the Philippines, respectively, in response to technical change in lowland rice. Lowland rice producers are price takers (they cannot influence world prices and take them as given; see box 1 for more details), and therefore they face an elastic excess demand for their product. They found that improving labour-intensive irrigation for lowland rice limited crop-land expansion in the uplands by drawing labour away from the forest margins. By contrast, the introduction of hand tractors led to land expansion in both the lowlands and upland forest in central Indonesia [41].

Over time, labour-saving and land-saving technologies that make agriculture more profitable can provide capital-constrained farmers with the surplus income needed to finance activities involving deforestation [1,22]. The case of double cropping (soybean followed by corn) in Brazil provides an interesting example. Double cropping increases the land endowment by allowing two annual harvests [21]. In the agricultural frontiers of the Brazilian Cerrado, land is abundant and cheap, while capital is often a primary limiting constraint. Double cropping in itself is capital-intensive as it requires large investments in inputs and machinery; double cropping in Brazil has also proven to be profitable. Richards & Arima [43] argue that these profit surpluses provide frontier farmers with the capital necessary to finance further land acquisition, infrastructure and speculative clearing. By effectively removing the capital constraint in an area with low land-conversion costs, this land-augmenting technology has resulted in considerable backfire rebound or Jevons effect [42].

The bias of technical change also conditions the magnitude of the rebound effects in subsistence settings, where an inelastic demand often leads to land sparing. In these environments, farm households are typically limited by capital and family labour rather than land. Consequently, the introduction of yield-enhancing or labour-intensive technologies acts as a ‘labour sink.’ For example, Fisher & Shively [49] found that providing subsidized seeds and fertilizer in Malawi increased the returns to farming, pulling household labour away from forest-degrading activities like charcoal production. Similarly, Abman *et al.* [46] found that an extension programme in Uganda promoting the use of intercropping and manure reduced deforestation by 14%. The authors hypothesize that increased labour demand in existing plots prevented them from clearing new land. Furthermore, by alleviating capital constraints and sustaining soil fertility, these yield-enhancing technologies removed the agronomic need for shifting cultivation, allowing farmers to meet their subsistence needs without continually abandoning degraded plots to encroach on the forest frontier [45,46].

## 4. Mitigating rebound effects

The examples above underscore the importance of sustaining agricultural productivity growth in subsistence settings where demand is inelastic. Pelletier *et al.* [65] show that smallholder adoption of improved maize seeds in Zambia, a landlocked, poorly market-integrated country, is significantly associated with reduced deforestation, illustrating that technology-driven yield growth can realize the land-sparing potential of inelastic demand. In a choice experiment, Vorlaufer *et al.* [66] find that Zambian smallholders are willing to commit to not clearing additional land in payment for environmental services schemes that reward conservation with in-kind payments rather than cash, even when the inputs are worth less than the cash alternative. They argue that the real reward lies in productivity gains that would not otherwise be obtained owing to poor access to input markets. In this sense, well-designed PES in smallholder settings can act as delivery mechanisms for the productivity investments that generate land sparing.

A mirror image to the land-sparing effects of productivity gains is provided by Hsiao *et al.* [67], who find that extreme heat-induced productivity declines cause large and persistent crop-land expansion and deforestation, with effects strongest precisely in regions with the most inelastic food demand. Taken together, these findings emphasize that investing in agricultural R&D and climate adaptation in subsistence-oriented, isolated settings contributes to both food security and land sparing.

As technological progress in tradable crops increases the profitability of agriculture, restraining rebound effects needs to be accompanied by spatial constraints such as land-use zoning, the creation of protected areas and rigorous satellite monitoring of forest clearing [1,68,69]. Institutional interventions such as strengthening property rights and recognizing indigenous common-use regimes can reduce the speculative land clearing often seen in frontier regions, thus constraining the land available for agriculture [3,68,70].

By constraining land through regulatory capping mechanisms, policymakers, effectively, artificially increase land scarcity, which can induce a shift towards intensification and innovation on existing plots [70,71]. The available evidence (focused on OECD countries) suggests that innovations that follow scarcity signals maximize total factor productivity growth [72]. This efficiency-driven growth allows the agricultural system to produce ‘more with less’, effectively using total factor productivity as a technological pressure valve that substitutes scarce natural resources with knowledge and innovative inputs [15,73]. In this sense, a policy environment that restricts the expansion of the agricultural frontier can act as a catalyst for innovation-driven productivity gains.

Market-based interventions can further steer these dynamics by taxing labour-saving mechanization—which often facilitates clearing at the frontier—while subsidizing labour-intensive intensification, such as technical irrigation, which pulls labour into existing high-quality plots [41,74]. New sources of demand add elasticity to global food markets, thus increasing the magnitude of backfire rebound effects locally, and reducing the land-sparing effects of technical change globally. An important source of the demand for corn as a second crop is the expansion of corn ethanol production. Jeddi & DePaula [42] propose that any fiscal support (tax incentives and rural credit) directed at the ethanol industry must be conditioned on verifying that corn meets zero land conversion standards.

Supply-chain initiatives such as market certification standards and commodity moratoria (e.g. the Soy Moratorium in Brazil) can be used to exclude produce grown on recently deforested land from global supply chains [1,64]. A recent evolution of supply chain interventions is the shift towards mandatory demand-side trade rules, most notably the European Union Deforestation Regulation (EUDR), which prohibits the import of key agricultural commodities unless they are strictly proven to be deforestation-free. Compliance with these regulations is most likely to succeed in export-oriented sectors like coffee, whereas sectors like cattle are highly challenging and may simply divert sales to less stringent alternative markets [75]. It should be kept in mind that the global effectiveness of these regulations is threatened by market leakage, such as the domestic displacement of Brazilian soy into unregulated biomes [76], the displacement of non-targeted crops to forest frontiers [77] or the diversion of soy exports from the EU to China [78]. Furthermore, indirect sourcing of commodities via local intermediaries creates a blind spot for traceability efforts, allowing non-compliant commodities to be easily laundered into seemingly deforestation-free supply chains [79]. These measures also involve high administrative costs of plot-level traceability that can exclude smallholder farmers from global supply chains [80]. In addition, because a narrow focus on plot-level traceability only addresses direct forest clearing and fails to capture indirect land-use change—such as crop expansion displacing cattle pasture into the forest frontier—these supply chain interventions must be complemented by broader jurisdictional governance to be truly effective [81].

## 5. Closing remarks

Agricultural productivity growth has been, on balance, a powerful force for land sparing. Between 2001 and 2010, technological progress avoided the conversion of an estimated 97 Mha of land to agriculture, with the most significant land savings occurring in the world's most biodiverse tropical and subtropical forests. This global land-sparing outcome is compatible with the seemingly paradoxical pervasiveness of domestic backfire rebound or Jevons effect once we take into account the direct and indirect land use effects of technological progress in different countries of the world. A critical question moving forward is whether we can sustain the conditions necessary to maintain this land-sparing trajectory. The sharp decline in agricultural TFP growth over the past decade and a half—from 2.00% annually in 2001–2010 to 1.17% in 2011–2020 and just 0.45% in 2021–2023—raises serious concerns. This slowdown stems from chronic underinvestment in public R&D, the mounting drag of climate change on yields, and persistent failures in technology transfer to developing regions where crop-land expansion remains the dominant source of output growth. Without a reversal of these trends, the historical land-sparing effects of technological progress cannot be taken for granted. Sustaining and accelerating productivity growth will require renewed commitment to agricultural R&D, particularly in climate adaptation, alongside the institutional and policy frameworks needed to ensure that efficiency gains translate into land conservation rather than further expansion at the frontier.

## Data Availability

This article has no additional data.

## Footnotes

^1^
The economic concepts used throughout are collected in box 1.

^2^
Thanks to a referee for bringing up this point.

^3^
95th percentile of observed regional yields, which represents a ‘feasible yield’, that generally remains 15–25% below the absolute agronomic potential.

^4^
Roberts & Schlenker [50] estimate that the aggregate global demand elasticity for calories derived from maize, wheat, rice and soybeans is extremely inelastic, ranging from –0.028 to –0.066, implying that a 100% increase in the price of a calorie derived from the included crops reduces demand by between 2.8 and 6.6 per cent. In their disaggregated four-crop system, they find individual own-price demand elasticities ranging from –0.244 to –0.287 for maize, –0.236 to –0.329 for soybeans and –0.095 to –0.109 for wheat. Similarly, Gouel & Legrand [56] report preferred price elasticities of demand of just –0.062 for maize and –0.033 for wheat.

^5^
Inelastic for food and feed uses; biofuel mandates and novel industrial uses may add elasticity at the margin (see §3.2).

^6^
Hertel *et al.* [16] formalize the roles of geographic scales, market shares and international trade in shaping the effects of agricultural technical change on crop-land outcomes by using the concept of excess demand elasticity discussed in box 1.

^7^
The sample composition in [62] was determined by data availability. Only 70 countries had complete data on agricultural TFP indices (ERS [25]), crop-land data from FAOSTAT and bilateral agricultural trade data. The 70 countries in the sample represent approximately 75% of worldwide cropland and account for 91% of global production, 86% of global imports and 91% of global exports.

^8^
[62] also reports instrumental variables (IV) estimates, using past public R&D expenditures to instrument for TFP growth, motivated by the fact that crop-land data used to construct the dependent variable also enters the TFP calculations, which could artificially inflate the estimates of area responses to TFP. The IV estimates were generally much larger and less precise than the OLS estimates, which provide more conservative estimates of the area responses to TFP.

^9^
As in the USA, productivity growth expands crop-land domestically in Canada and Argentina; but these countries' smaller weights in world markets render the associated land savings abroad insufficient to offset their domestic expansion, producing a net increase in global crop-land [62].
